# Update on a previously reported missense mutation: The c.1160 C>A mutation in the *UGT1A1* gene result in Crigler–Najjar syndrome type 1

**DOI:** 10.1002/mgg3.1805

**Published:** 2021-09-21

**Authors:** Mohammad Javad Ghorbani, Seyed Mohsen Dehghani

**Affiliations:** ^1^ Neonatal Research Center Shiraz University of Medical Science Shiraz Iran; ^2^ Gastroenterohepatology Research Center Nemazee Teaching Hospital Shiraz University of Medical Sciences Shiraz Iran

## CONFLICT OF INTEREST

The authors declare that there is no conflict of interest.

## AUTHOR CONTRIBUTIONS

Clinical studies and study design performed by Seyed Mohsen Dehghani. The experiments, analyzing the data, and manuscript preparation were performed by Mohammad Javad Ghorbani. All authors have read and approved the final manuscript.

## ETHICAL COMPLIANCE

This study was approved by the ethics committees of Shiraz University of Medical Sciences: IR.SUMS.REC.1399.252.

To the Editor,

Crigler–Najjar syndrome (CN) is a rare inherited disorder with a frequency of one per million. CN is characterized by non‐hemolytic unconjugated hyperbilirubinemia. Unconjugated hyperbilirubinemia is caused by the limitation or absence of bilirubin uridine 5′‐diphosphate glucuronosyltransferase (UGT1A1) enzyme activity. UGT1A1 converts unconjugated bilirubin into conjugated bilirubin (water‐soluble), which is essential for bilirubin excretion.

CN has two types based on clinical criteria and phenobarbital response. CN type I (CN‐I; MIM# 218800) is the most severe form of CN. Patients with CN‐I have severe hyperbilirubinemia and usually die due to kernicterus. The absence of the UGT1A1 enzyme activity due to a defect in the *UGT1A1* gene (UGT1A1; MIM# 191740) result in CN‐I (Gailite et al., 2020). Liver transplantation is the only definitive therapy for CN‐I (Lysy et al., 2008). CN type II (CN‐II; MIM# 606785) is less severe and less likely to develop kernicterus (Liaqat et al., 2018). Patients with CN‐II have residual UGT1A1 enzyme activity. Serum bilirubin levels can usually be lowered by treatment with phenobarbital in CN‐II (Canu et al., 2013). However, phenobarbital has no role in CN‐I. Phenobarbital increases the expression of UGT1A1, but that will only be effective if the mutated enzyme has residual activity (Sugatani et al., 2001).

**FIGURE 1:**
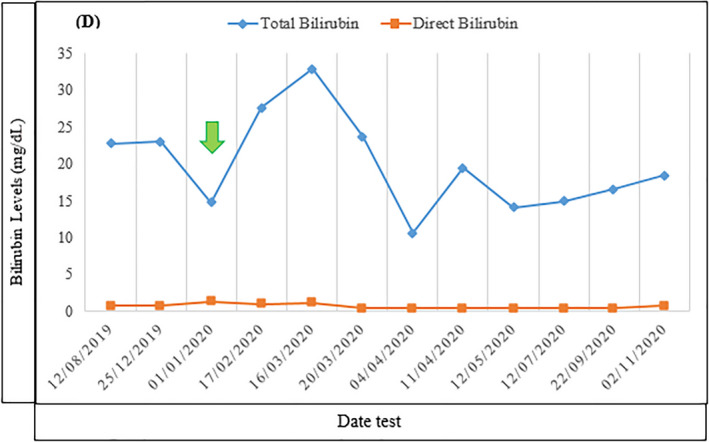
Total and direct bilirubin levels are shown with the date of the test. The date of phenobarbital administration is indicated by the green arrow

A 14‐month‐old boy with persistent unconjugated hyperbilirubinemia was referred to the Abu Ali Sina Transplant Hospital, Shiraz, Iran (the main center of liver transplantation in Iran) for a definite diagnosis of CN. However, the patient was treated repetitively with phototherapy. Unconjugated bilirubin levels were elevated with advancing age. Furthermore, unconjugated bilirubin concentration did not decrease significantly after phenobarbital administration (Figure 1). The phenobarbital response was tested by oral administration of 7 mg/kg/day. The patient had no history of neonatal viral infections, red blood cell (RBC) enzyme abnormality, hematoma, hemolysis, rash, hypothyroidism, and all other common causes of jaundice. Based on the criteria of CN phenotypes adapted from Fabris et al. (2009), the diagnosis of CN‐I was made for the patient. We evaluated the promoter and all five exons of the human *UGT1A1* gene (NG_009254) to confirm the diagnosis. Informed consent (approved by the ethics committees of Shiraz University of Medical Sciences: IR.SUMS.REC.1399.252) was signed by parents.

**FIGURE 2:**
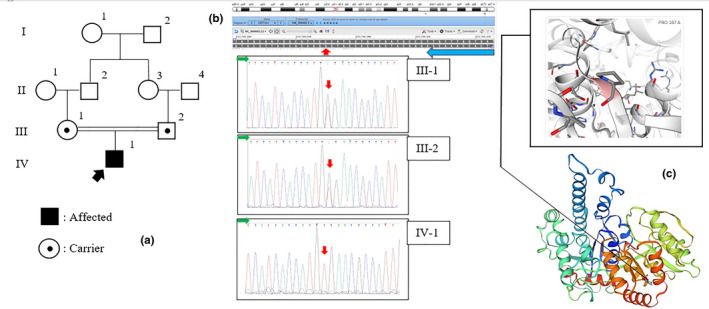
Pedigree, electropherogram, and protein structure. (a) The pedigree. The proband is indicated by a black arrow. (b) The electropherograms of the family members compared with sequences that were taken from the NCBI (National Center for Biotechnology Information). The location of the mutations is indicated by a red arrow and the direction of reading in the electropherogram and reference sequences is shown by a green and blue arrow, respectively. (c) Structure analysis of the UGT1A1 protein showed that the p.P387 is an active site

We identified a missense mutation (NM_000463.3: c.1160 C>A; p.P387H) in the homozygous state in exon 4 of the *UGT1A1* gene in the proband. Extensive sequencing of the remaining allele did not reveal any other structural mutations. The c.1160 C>A variant was present in the heterozygous state in the parents (Figure 2a,b). The UGT1A1 protein structure analysis showed that the p.P387 is one of the active sites of the UGT1A1 enzyme (Figure 2c). Based on the American College of Medical Genetics and Genomics (ACMG) variant classification guidelines (Richards et al., 2015), the c.1160 C>A mutation is a pathogenic variant (PS1, PM1, PM5, PP2, PP3, and PP4).

Previously, Sneitz et al. reported the c.1160 C>A variant in the heterozygous state in a CN‐II patient as a novel mutation in the article entitled: “Crigler‐Najjar Syndrome in The Netherlands: Identification of Four Novel *UGT1A1* Alleles, Genotype–Phenotype Correlation, and Functional Analysis of 10 Missense Mutants” (Sneitz et al., 2010). Sneitz et al. (2010) showed that the c.1160 C>A mutation did abolish the activity of the enzyme. The mutation was identified in a CN type II patient in a heterozygous state. The residual UGT1A1 enzyme activity in this patient responsible for the phenotype was encoded by the other allele. Now we report that homozygosity for this mutation indeed causes CN‐I, confirming the complete inactivity of this mutant. Genotype–phenotype correlation analysis of the c.1160 C>A mutation showed that this mutation is pathogenic in the homozygous state and leads to CN‐I. Our findings would be beneficial for the clinicians to diagnose CN‐I. Liver transplantation is the only curative treatment of CN‐I. Accordingly, timely diagnosis, can improve patient prognosis. Also, the molecular diagnosis might enable the family of patients to perform prenatal diagnosis and prevention.

## Data Availability

All data generated or analyzed during this study could be obtained from the authors upon reasonable requirements.
